# Candida score: a predictor of mortality in patients with candidemia

**DOI:** 10.1186/cc11704

**Published:** 2012-11-14

**Authors:** D Juneja, P Nasa, O Singh, Y Javeri, S Garg

**Affiliations:** 1 Max Super Speciality Hospital Saket, New Delhi, India

## Background

Candidemia is becoming increasingly widespread, especially in the ICU setup. The Candida score has been developed and used for identifying patients at risk for developing candida infections. However, its usefulness in predicting outcome of patients with candidemia has not been evaluated. We aimed to determine the risk factors for mortality in patients with candidemia admitted to an Indian medical ICU.

## Methods

An 18-month retrospective cohort analysis including 56 patients with candidemia was conducted. Baseline patient characteristics, ICU course and outcome were noted in a predesigned *pro forma*. The Candida score was calculated as previously described [1]. The primary outcome measure was ICU mortality.

## Results

Out of 3,142 ICU admissions, the incidence of candidemia was 17.8/1,000 admissions. Fifteen patients had co-existing candiduria with the same species. The mean interval between ICU admission and candidemia was 12.9 ± 14.4 days. *Candida albicans *was isolated in only 21.4% of blood *Candida*-positive cultures. Among the nonalbicans species *C. tropicalis *was the commonest species isolated from 28.6% isolates, followed by *C. glabrata *that was isolated from seven (12.5%) patients. In 8.9% of candidemia patients, the only measure taken was removal of the indwelling catheter, but the rest of the patients required antifungal drugs. Among the patients with candidemia, 53.6% required vasopressor support, 41.1% required renal replacement therapy and 64.3% required mechanical ventilation during their ICU stay. The mean length of ICU stay was 22.9 ± 28 days and the mean hospital stay was 30.1 ± 30.2 days. Crude ICU mortality was 33.93%. There was no statistically significant difference between mortality rates of patients with *C. albicans *and nonalbicans candidemia (*P *= 0.732). Patient parameters such as age, admission APACHE II score, candida score, previous antifungals and underlying co-morbidities, which were statistically significant in differentiating survivors and nonsurvivors in the univariate analysis (Table 1), were included in the multivariate analysis. Only two factors, previous antifungals (*P *= 0.004, OR = 101.4, 95% CI = 4.52 to 227.7) and Candida score >3 (*P *= 0.028, OR = 13.2, 95% CI = 1.3 to 125) were found to be independently predicting mortality.

**Table 1:** Comparison patient parameters among survivors and nonsurvivors

Parameter of interest	Overall (*n *= 56)	Survivors (*n *= 37)	Nonsurvivors (*n *= 19)	*P *value
Age, mean	63.07 ± 20.7	59.11 ± 21.9	70.79 ± 16.1	0.045*
Sex, males (%)	41 (73.2%)	27 (73%)	14 (73.7%)	0.955
APACHE II score	19.38 ± 7.7	17.32 ± 7.6	23.37 ± 6.2	0.004*
Predicted death rate (%)	36.87 ± 22.6	31.05 ± 21.7	48.19 ± 20.2	0.006*
Nonalbicans infection	44 (78.6%)	31 (83.8%)	13 (68.4%)	0.302
Co-existing candiduria	15 (26.8%)	10 (27%)	5 (26.3%)	0.955
Underlying co-morbidities	41 (73.2%)	24 (64.9%)	18 (94.7%)	0.021*
Previous antimicrobials	49 (87.5%)	33 (89.2%)	16 (84.2%)	0.679
Previous antifungals	8 (14.3%)	1 (0.3%)	7 (36.8%)	0.001*
Days in ICU before positive culture	12.88 ± 14.4	13.05 ± 14.6	12.53 ± 14.3	0.898
Recent hospitalization	17 (30.4%)	11 (29.7%)	6 (31.6%)	0.887
Total parenteral nutrition	18 (32.1%)	15 (40.5%)	3 (15.8%)	0.076
Candida score (≥3)	29 (51.8%)	15 (40.5%)	14 (73.7%)	0.025*
Presence of CVC	49 (87.5%)	31 (83.8%)	18 (94.7%)	0.403
Mechanical ventilation	36 (64.3%)	19 (51.4%)	17 (89.5%)	0.007*
Vasopressor support	30 (53.6%)	13 (35.1%)	17 (89.5%)	0.000*
Renal replacement therapy	23 (41.1%)	9 (24.3%)	14 (73.7%)	0.001*
Length of ICU stay (days)	22.86 ± 28	22.35 ± 31.4	23.84 ± 20.6	0.853
Length of hospital stay (days)	30.09 ± 30.2	33.19 ± 33.8	24.05 ± 21.1	0.288

*Statistically significant.

## Conclusion

Candida infection is generally late-onset in ICU patients and is associated with a prolonged ICU and hospital stay, and a high mortality. Candida nonalbicans infection was much more common in our cohort of ICU patients but there was no difference in mortality among patients with *C. albicans *and nonalbicans infection. Patients who develop candidemia, despite being on antifungals, were at a higher risk of dying and a simple bedside candida score may be useful in predicting mortality of ICU patients with candidemia.
